# When should the external approach be resorted to in the arthroscopic treatment of perimeniscal cyst?

**DOI:** 10.1051/sicotj/2015046

**Published:** 2016-04-20

**Authors:** Hasan Bombaci, Mehmet Kuyumcu, Tamer Coskun, Emre Kaya

**Affiliations:** 1 Haydarpasa Numune Education and Research Hospital, Orthopaedics and Traumatology Department Uskudar, Istanbul Turkey; 2 Hacettepe University, Hand Surgery Department, Hacettepe Ankara Turkey; 3 Camlıca Erdem Hospital Uskudar, Istanbul Turkey

**Keywords:** Knee, Meniscal cyst, Meniscectomy, Arthroscopy

## Abstract

*Introduction*: Meniscal cysts very often cause meniscal tears and especially when it is peripheral, some of the healthy parts of meniscus might be needlessly sacrificed. In particular conditions, extraarticular approaches might save some menisci. In the present study, we evaluated the conditions which required using the extraarticular approach in addition to the arthroscopic procedure, to maximally preserve the meniscus.

*Methods*: Eight patients with perimeniscal cysts were evaluated retrospectively. One cyst was localized within the medial meniscus and seven in the lateral meniscus. The mean age was 36.13 (range; 19–63) years, mean follow-up time, 27.3 (range; 12–47) months. Patients were evaluated by using a Visual Analogue Score (VAS) to measure pain relief and “Lysholm score” to measure functional improvement. In all patients except one, in which the cystic cavity was connected with the joint at the periphery of the meniscus, the cyst was drained from the intraarticular opening. When the cyst was too large (three cases) and in one case where a large amount of meniscus was preserved for reasons mentioned above, additional extraarticular drainage was carried out.

*Results*: The mean preoperative and postoperative VAS were 6 (range; 2–8) and 1.55 (range; 0–3) (*p* = 0.00058) and Lysholm scores were 64.75 (range; 48–86) and 93.11 (range; 80–100) (*p* = 0.0014), respectively.

*Discussion*: In cysts, which have very limited or no connection with the joint on the most peripheral region of the meniscus and/or are larger than the meniscus height, extraarticular drainage of the cyst might produce unnecessary meniscal loss and function. In the extraarticular drainage, scrapping the walls of the cyst, while inspecting with an arthroscope, reduces recurrence of the cyst.

## Introduction

Perimeniscal cysts are mostly formed in the lateral meniscus and cause meniscal tears. In particular, the ones which are formed in the degenerated menisci are, tiny intrameniscal cysts very often encountered on the magnetic resonance images (MRI). Formerly, the preferred treatment method of a meniscal cyst was open drainage with/without total surgical meniscectomy, but recently after the advent of arthroscopic techniques, various treatment methods can be used. Because of long-term poor results after meniscectomy, as meniscus sparing methods replaced meniscectomy, the same tendency is becoming more popular in the tears related to the meniscal cyst [1–3]. As a drainage procedure in some cases arthroscopic technique is sufficient however in other cases, an open technique used depending on the location of the cyst and the amount of meniscal damage developed [3–5]. More recently, minimal resection of the meniscal tears related to an intrameniscal cyst and suture of the tear in suitable cases are used increasingly more often [2]. Although there are some studies which reveal the negative effects of partial and total meniscectomy, the long-term outcome of cases with meniscal cyst, which are treated by partial meniscectomy, have not been reported yet [1].

The purpose of this study, which includes cysts in the relatively rare location such as posterior horn of the lateral meniscus and medial meniscus, is to determine the clinical outcomes of meniscal tears related to the cyst, and to discuss our indications for resorting to the extraarticular approach in the cases where arthroscopic procedures were used. It was hypothesized that to resort to the extraarticular approach in the relevant cases provides much less resection of the damaged meniscus and achieves comparable clinical outcomes when the arthroscopic technique is used alone.

## Materials and methods

Between 2008 and 2011, eight (six male, two female) of 230 knee arthroscopies in 223 patients, who were operated on by one surgeon, had a meniscal cyst. These cases were evaluated retrospectively. In the present study, “ethical principles for medical research involving human subjects” were followed. The patients were informed that data from the case would be submitted for publication and gave consent. The mean age was 36.13 (range; 19–63) years, mean follow-up time, 27.3 (range; 12–47) months. Right knee was involved in six patients, left knee, in two. There was no additional surgery or conservative treatment other than the index surgical procedure for the cyst. The common complaint was pain, which was localized on the meniscal cyst as determined by the MRI findings. Three of them had an additional a “clicking sign”. The pain became more intense with flexion or extension of the knee dependent upon the location of the cyst. Five of the patients (62.5%) had a previous history of trauma but three (37.5%) did not. The mean interval between symptoms first appearing and operation was 40 months (range; 1 month–10 years). When the patients were assessed according to the “body mass index” (BMI), four cases were evaluated as “normal” (18.5–24.9), and four, “overweight” (25–29.9).

**Figure 1.**
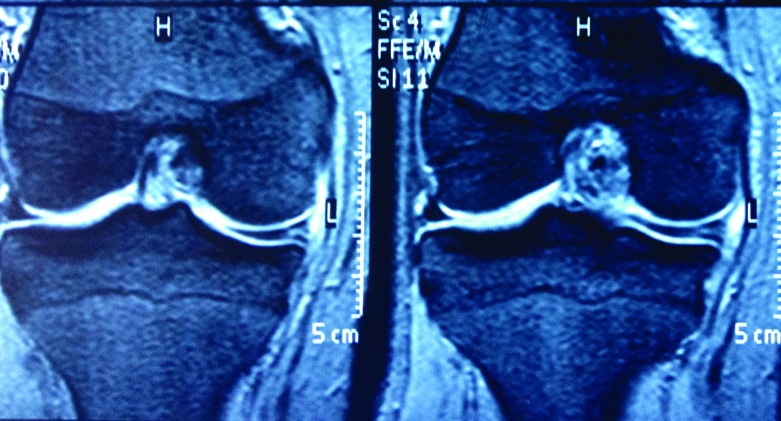
Preoperative coronal magnetic resonance (MR) image of the right knee. Small horizontal tear is seen on the peripheral meniscus. Larger part of meniscus is intact on the central part.

**Figure 2.**
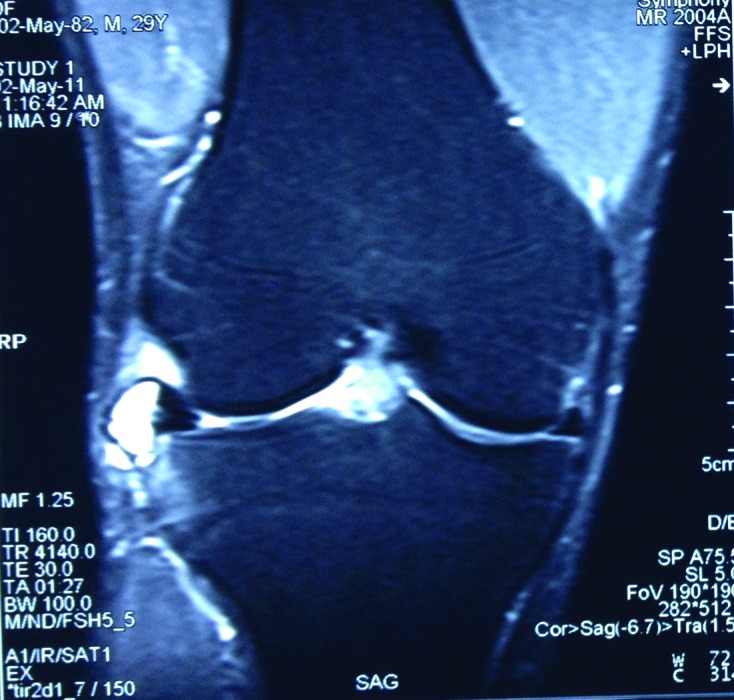
Perimeniscal cyst is seen on the middle horn of lateral meniscus of right knee. Horizontal tear, which extends along the entire meniscus, is seen on the preoperative coronal MR images.

**Figure 3.**
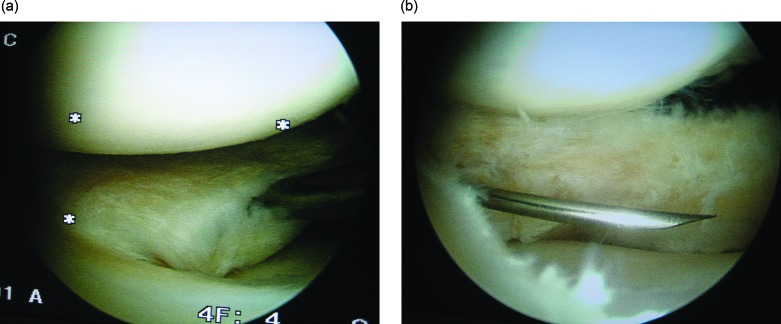
(a) Cyst is seen on the middle horn of lateral meniscus. Intraoperative arthroscopic view of the cyst before partial meniscectomy; (b) after partial meniscectomy. The needle, which passes through the horizontal tear left after partial meniscectomy, indicates the place where the extraarticular procedure will be added in the next section.

Arthroscopy was performed in all patients with standard anteromedial (AM) and anterolateral (AL) portals. Outerbridge classification was used to describe associated cartilage lesions. In the beginning of the operation, the place of cystic cavity, which was determined with the help of MRI before the operation, was marked with 21 gauge needle. In all patients except one, in which the cavity was connected with the joint at the very periphery of the meniscus, the cystic cavity was drained from the intraarticular opening. When the cyst was too large (larger than the meniscus height) (three cases), and in one case where the meniscus was largely preserved as mentioned above, additional drainage was used using an extraarticular incision (Figures 1 and 2). In the first three cases, decompression was carried out through “an extraarticular portal”, however in the latter case, “mini-open access” was used without penetrating the joint cavity. The cystic cavity was delicately scraped with a curette and/or a 3.5 mm shaver. To be sure of being inside the cyst during scrapping, the meniscus was inspected by arthroscopy to ascertain whether the curette or shaver tented the wall of the cyst in applicable cases. In seven cases, in which the horizontal tear reached the inner 1/3 of the meniscus, the unstable leaves of the horizontal meniscal tear were excised until the stable and vascularised peripheral portion, up to outer 1/3 of the meniscus was reached. During this procedure it was attempted to preserve as much meniscus as possible. This peripheral portion of the meniscus is more resistant to the mechanical stresses and has healing potential (Figure 3). In the postoperative period, immediate weight bearing was allowed. Patients resumed daily activities of living as much as the pain permitted.

Before surgery and at the last review, patients were evaluated by using VAS to measure pain relief which is a common complaint in all patients, and “Lysholm score” to measure functional improvement. The results were analyzed statistically by using paired *t* test, *p* value < 0.05 was accepted as significant.

## Results

**Figure 4.**
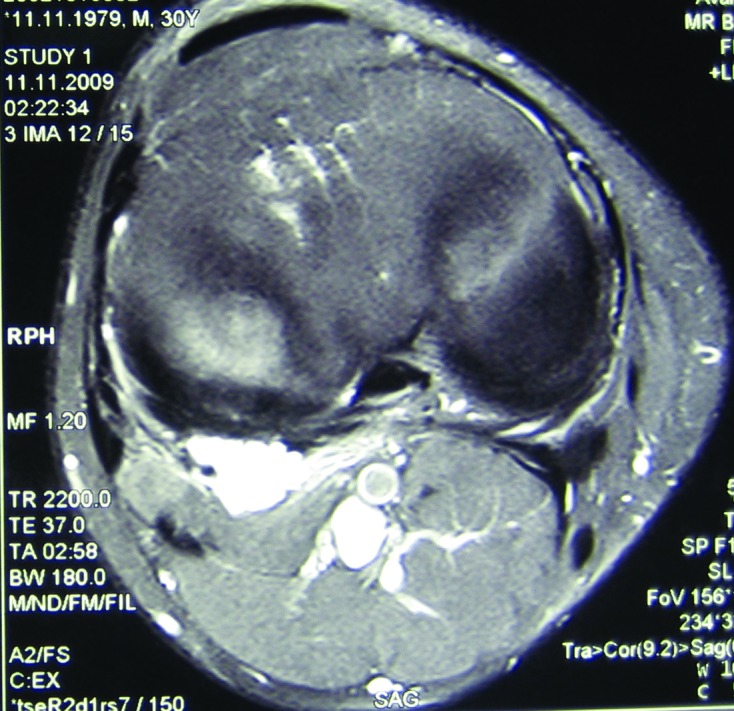
Perimeniscal cyst is seen on the axial MR imaging of the posterior horn of the lateral meniscus of right knee.

One of the cysts was in the medial meniscus, seven in lateral meniscus at different localizations (Table 1). The one which involved the posterior third of the lateral meniscus and the one that involved the middle third of the medial meniscus were quite rare localizations (Figure 4) [6]. In seven of the eight cases, there was a horizontal tear which extended nearly the full-width of the meniscus and opened into the joint. Some of the patients had additional cartilage lesions, which were classified as I to III according to the Outerbridge classification. No procedure was performed for the cartilage lesions encountered.

**Table 1.** Localization of the perimeniscal cysts.

	Anterior horn	Middle horn	Posterior horn	Total
Medial meniscus	0	1	0	1
Lateral meniscus	0	6	1	7
Total	0	7	1	8

The mean preoperative and postoperative VAS were 6 (range; 2–8) and 1.55 (range; 0–3), respectively. Lysholm scores were 64.75 (range; 48–86) and 93.1 (range; 80–100), respectively. When the preoperative and postoperative values were compared statistically, *p* values (0.0058 and 0.0014, respectively) were very significant. The preoperative mean Lysholm score of the patients treated with extraarticular procedure in addition to arthroscopy (52.75 ± 4.113) was lower than that of the patients (76.75 ± 7.182) treated with the arthroscopic technique alone (*p* = 0.007). However, the postoperative scores were similar in the two groups (respectively; 95.25 ± 6.602 and 94.25 ± 7.805) (*p* = 0.8671). While the difference between preoperative (52.75 ± 4.113) and postoperative Lysholm scores (95.25 ± 6.602) was very significant in the patients treated with the arthroscopy and extraarticular procedure (*p* = 0.0002), it was not significant in the patients treated with the arthroscopy alone (respectively; 76.75 ± 7.182 and 94.25 ± 7.805) (*p* = 0.0993).

At the last follow-up, six patients had an MRI. One MRI revealed recurrence of the cyst with much smaller dimensions. In this case, the cyst was extending along more than two thirds of the meniscus with intralesional septa formation before surgery. In the other patient, there was a tiny cyst at the periphery of meniscus without any symptom. In the remaining four patients, no cysts were found on MRI.

## Discussion

The findings of this study suggest that, the outcomes of extraarticular drainage, in addition to arthroscopic technique, of the meniscal cyst, which are larger than meniscus height, might be as good as the smaller ones treated with the arthroscopic technique alone.

The incidence of the lateral meniscus cyst is higher compared to that of the medial meniscus cyst [5–9]. On the lateral meniscus, posterior localization is not seen quite so often [6, 10, 11]. Medial meniscus cysts are even rarer. Hulet et al. reported a large series of 8100 cases and found 124 lateral meniscus cysts compared with one medial meniscus cyst [9]. In this series, there was also only one medial meniscus cyst. The incidence rate of medial meniscus cyst in this series (4.34%) is comparable with the rate of Mills and Henderson’s series (4.49%) [12].

Hulet et al. reported that pain at the joint line was a common complaint in all patients, and in some cases pain was associated with another sign (i.e. hydrarthrosis) [9]. In the present series, the most frequent complaint, secondary to pain, was “clicking sign” on the joint. The pain was peculiar with being more intense by increased flexion or extension of the knee dependent upon localization of the cyst. The reason for the more frequent “clicking sign” in the present series might be related to most of the patients (62.5%) having a history of trauma. Hydrarthrosis, which is a frequently associated finding in the Hulet’s series, is not the foremost complaint in this series [9]. It might be related to a higher incidence of grade 3 or 4 chondropathy in any of the three compartments of the knee joint in Hulet’s series (63%), compared with this series (12.5% – one patient) [9].

There are some studies which reveal the relations between the BMI and osteoarthritis; however, there is no study which compares the BMI and meniscal lesions [13]. When the cases, in the present series, were analyzed from this point of view, the BMI of half of the patients was above the normal range. This finding must be considered prudently, because of the limited number of cases and the absence of a control group.

Arthroscopic partial meniscectomy of the involved part of meniscus and intraarticular cyst drainage has become the most accepted procedure in perimeniscal cysts [9, 14]. Hulet et al. stated that because a meniscal tear results from a degenerative breakdown of the meniscal collagen, meniscectomy, as much as the size of the cyst entails, might prevent recurrence. The author also claims that excessive conservative resection of the meniscus results in the need for reoperation [9]. In our opinion, the crucial issue to prevent recurrence is to eliminate the valve mechanism. It is claimed that either traumatic or degenerative, the influx of synovial fluid through microscopic and macroscopic tears in the substance of the meniscus causes enlargement of the cyst and subsequently development of meniscal tears [6, 15]. In our opinion, in the cases in which the meniscal tear clearly opens into the joint cavity, meniscectomy should be limited to the unstable part of the tear. If the tear is not opened into the joint, arthroscopy should not go on further after the meniscus examination. However, even in the cases mentioned later, the valve mechanism should be addressed [16]. For this reason in this series, after the limited meniscectomy in the applicable cases, a hole is created through the preserved meniscus, from the apex of the cleavage to the very peripheral wall of the cystic cavity. In this way, the valve mechanism which is proposed for meniscal cyst is eliminated and an adequate passage is designed between the cyst and the joint cavity. So, it was expected the pressure in the cystic cavity to lower and symptoms to diminish. In our opinion, this is the main reason for the improved VAS and clinical results in this series at a mean of 2.7 year follow-up period (*p* = 0.0058 and *p* = 0.0014, respectively). We also believe that, although a degenerated meniscus is prone to the formation of meniscal cyst, as long as it is stable, it may carry on to contribute load sharing on the joint. Even the cyst formation is unavoidable; it is not known how much time is required for a cyst to become symptomatic. Therefore recently, resection of the partly damaged meniscal tissue, which was developed after perimeniscal cyst, has been avoided as much as possible [2].

Horizontal tears of the meniscus, which propagates from the periphery of the meniscus after formation of the intrameniscal cyst, sometimes extend as far as most inner parts of the meniscus. Some authors reported good results with only arthroscopic debridement or partial meniscectomy; however, Reagan et al. suggested additional extraarticular drainage is needed [3–5, 11, 17]. We agree with Reagan et al. In the present series, we changed our treatment approach according to the location of the horizontal tear opening into the joint cavity and the size of the cyst [3]. In all cases, the arthroscopic technique was used, but in one case the meniscus tear was very short and was opening into the joint at the very far peripheral region. The cystic cavity was reached via extraarticular approach and drainage was successful as well as preserving the healthy meniscus. The inner wall of cyst was also scraped with the shaver. This might theoretically allow revascularization in the blood-rich region. If this procedure is done by reaching through the meniscus instead of the extraarticular way, it might cause damage to the rest of meniscus, especially where there is larger cyst. Also we believe that, the excision of a horizontal tear in the innermost avascular part of the meniscus might increase the resistance of the remaining meniscus and allow a decrease of stresses subsequently increasing healing of the degenerated part. Our findings support this claim. In the present series, more significant improvement of clinical outcomes was obtained in the cases with a larger cystic cavity in which an extraarticular procedure was used (*p* = 0.0002).

Moreover, Mills and Henderson stated that the cystic cavity of the meniscus is lined with a synovial type of epithelium [12]. Therefore, we believe that, scraping the cyst wall with a shaver accelerates the healing of the remaining meniscus by increasing revitalization. In the cysts which are localized to the peripheral meniscus, the wall of the cavity can also be cauterized. However, we believe that making the sole vascular zone of the meniscus avascular might slow the healing process and increase the recurrence rate. For this reason, in the present study, the wall of the cavity has been revitalized with a shaver in this manner, therefore the healing potential of the remaining meniscus and cyst has been increased. We believe that this is the main reason for only one recurrence in the five cases examined with a control MRI. However, the very large cysts, which extend along almost two thirds of the meniscus with multilobular formation, still have a higher risk of recurrence as the single case in this series.

This study has some limitations. Firstly, the number of patients is limited. However symptomatic intrameniscal cysts have not been encountered frequently and this study includes a quite rare localization of a meniscal cyst (i.e. medial meniscus and posterior horn of lateral meniscus). Secondly, some of the patients had additional cartilage lesions. However, cartilage lesions are not rare findings in the patients presenting with a symptomatic meniscal cyst. No procedure for the cartilage lesion, which might affect the outcomes, was performed in this series. Thirdly, the follow-up time (mean 27.3 months) is not long when it is considered that the meniscal cyst is a slow developing lesion. However, the minimum period of time for the meniscal cyst to become symptomatic although of eight patients, six had an MRI which showed no recurrence. Unfortunately, it is sometimes hard to convince the asymptomatic patient to have a further examination.

## Conclusion

In meniscal tears, which developed from the perimeniscal cyst or degenerated meniscus tissue and opened into the joint cavity, partial meniscectomy, which was performed by preserving the peripheral meniscus, enhances the healing of tears in the remaining meniscus. In cysts, which are larger than the height of the meniscus, extraarticular drainage is additionally recommended. In the extraarticular drainage, scraping the walls of the cyst, while inspecting with an arthroscope, reduces the chance of a recurrence of the cyst. In the cysts, which do not open into the joint cavity or have very limited connection with the joint on the most peripheral region of meniscus, extraarticular drainage of the cyst without meniscectomy might produce unnecessary meniscal loss and function without improving the risk of recurrence.

## Conflict of interest

The authors declare that they have no conflict of interest.
